# Measuring self and informant perspectives of Restricted and Repetitive Behaviours (RRBs): psychometric evaluation of the Repetitive Behaviours Questionnaire-3 (RBQ-3) in adult clinical practice and research settings

**DOI:** 10.1186/s13229-024-00603-7

**Published:** 2024-06-06

**Authors:** Catherine R.G. Jones, Lucy A. Livingston, Christine Fretwell, Mirko Uljarević, Sarah J. Carrington, Punit Shah, Susan R. Leekam

**Affiliations:** 1 Wales Autism Research Centre, School of Psychology, Cardiff University, Cardiff, UK; 2 Cardiff University Centre for Human Developmental Science, School of Psychology, Cardiff University, Cardiff, UK; 3 Department of Psychology, Institute of Psychiatry, Psychology and Neuroscience, King’s College London, London, UK; 4 Integrated Autism Service, Aneurin Bevan University Health Board, Pontypool, Wales, UK; 5 Department of Psychiatry and Behavioural Sciences, Stanford University, Stanford, CA US; 6 School of Psychology, College of Health and Life Sciences, Aston University, Birmingham, UK; 7 Department of Psychology, University of Bath, Bath, UK

**Keywords:** Autism, Validity, Self-report questionnaire, Clinical service, Restricted and repetitive behaviours, RBQ-3, DISCO

## Abstract

**Background:**

Brief questionnaires that comprehensively capture key restricted and repetitive behaviours (RRBs) across different informants have potential to support autism diagnostic services. We tested the psychometric properties of the 20-item Repetitive Behaviours Questionnaire-3 (RBQ-3), a questionnaire that includes self-report and informant-report versions enabling use across the lifespan.

**Method:**

In Study 1, adults referred to a specialised adult autism diagnostic service (*N* = 110) completed the RBQ-3 self-report version, and a relative or long-term friend completed the RBQ-3 informant-report version. Clinicians completed the abbreviated version of the Diagnostic Interview for Social and Communication Disorders (DISCO-Abbreviated) with the same adults as part of the diagnostic process. For half of the assessments, clinicians were blind to the RBQ-3 ratings. We tested internal consistency, cross-informant reliability and convergent validity of the RBQ-3. In Study 2, a follow-up online study with autistic (*N* = 151) and non-autistic (*N* = 151) adults, we further tested internal consistency of the RBQ-3 self-report version. We also tested group differences and response patterns in this sample.

**Results:**

Study 1 showed good to excellent internal consistency for both self- and informant-report versions of the RBQ-3 (total score, α = 0.90, ω = 0.90, subscales, α = 0.76-0.89, ω = 0.77-0.88). Study 1 also showed cross-informant reliability as the RBQ-3 self-report scores significantly correlated with RBQ-3 informant-report scores for the total score (*r*^*s*^ = 0.71) and subscales (*r*^*s*^= 0.69-0.72). Convergent validity was found for both self and informant versions of the RBQ-3, which significantly correlated with DISCO-Abbreviated RRB domain scores (*r*^*s*^ = 0.45-0.54). Moreover, the RBQ-3 scores showed significantly weaker association with DISCO -Abbreviated scores for the Social Communication domain, demonstrating divergent validity. Importantly, these patterns of validity were found even when clinicians were blind to RBQ-3 items. In Study 2, for both autistic and non-autistic groups, internal consistency was found for the total score (α = 0.82-0.89, ω = 0.81-0.81) and for subscales (α = 0.68-0.85, ω = 0.69-0.85). A group difference was found between groups.

**Limitations:**

Due to the characteristics and scope of the specialist autism diagnostic service, further testing is needed to include representative samples of age (including children) and intellectual ability, and those with a non-autistic diagnostic outcome.

**Conclusions:**

The RBQ-3 is a questionnaire of RRBs that can be used across the lifespan. The current study tested its psychometric properties with autistic adults without intellectual disability and supported its utility for both clinical diagnostic and research settings.

**Supplementary Information:**

The online version contains supplementary material available at 10.1186/s13229-024-00603-7.

## Introduction

Restricted and repetitive behaviours (RRBs) form a complex domain of behaviours that span a wide range of different presentations including motor stereotypies, sensory features, routines, and special interests. They are characterised by high levels of repetition, narrowness of focus and/or intense preference for sameness [1]. RRBs are widely found in the general population [2] and across many neurodevelopmental and neuropsychiatric conditions [3–5]. However, they are particularly important for autism diagnosis, being one of the two diagnostic domains defined by the DSM-5 criteria for autism spectrum disorder (ASD) [6].

### Assessment of restricted and repetitive behaviours

Best-practice clinical assessment of autism typically requires a detailed interview, behavioural observation and assessment of needs according to DSM-5 [6] or the International Classifications of Diseases (ICD-11); [7]. Recently, brief questionnaires have been introduced that offer the potential for streamlined information-gathering before or alongside standardised diagnostic methods [8, 9]. While the most widely used general screening and/or quantitative measurement questionnaires such as the 10-item Autism Spectrum Quotient (AQ-10; 10), the Social Communication Questionnaire (SCQ); [11] and Social Responsiveness Scale (SRS); [12] capture broad autism traits, they have limited content coverage of RRBs [13]. Given that RRBs are required for diagnosis, a questionnaire that includes a broad range of RRBs can help to guide a clinician before their diagnostic assessment of an individual. RRB questionnaires provide information about the frequency, type and intensity of particular behaviours that are relevant for that individual, and assist the clinician in focusing on and exploring those items when they carry out diagnostic interviews and observations. Examples of such measures include, the Repetitive Behavior Scale-Revised (RBS-R; [14]), the Childhood Routines Inventory-Revised (CRI-R; [2]) and Adult Routines Inventory (ARI; [2]), the Dimensional Assessment for Restricted and Repetitive Behaviours (DARB); [15] and the Repetitive Behaviours Questionnaire-2 (RBQ-2; [16]) and − 2 A (RBQ-2A; [17]).

There is evidence that these dedicated RRB questionnaires have high levels of measurement precision [18] as well as acceptable psychometric properties [19]. However, currently, these questionnaires are limited by the fact that they have been validated as either self-report or informant-report measures and are used with specific age groups. This significantly limits their utility for lifespan capture of RRBs. A questionnaire with both self- and informant-report versions is required for applicability across the spectra of age and cognitive abilities, from those with intellectual disability [6] to ability in the normative range. This will enable more streamlined clinical practice, more inclusive research, and broaden opportunities for longitudinal life-span research. It is also important to recognise that the reach of RRB questionnaires has the potential to extend beyond autism-focussed clinical practice and research. There is growing awareness that RRBs are found dimensionally within the general population [16, 17], where they are associated with emotional and behavioural difficulties [20, 21], and can also be notable in other clinical populations [22, 23].

The Repetitive Behaviours Questionnaire-3 (RBQ-3) is a new 20-item measure that consolidates the previous RBQ-2 [16] and RBQ-2A [17] questionnaires, providing a coordinated pair of self-report and informant-report versions for use across the lifespan. For individuals who cannot use self-report, for example, young children and individuals with significant intellectual disability, the informant-report version can be completed as a stand-alone questionnaire by parents, guardians or support workers. For other individuals, including older children, adolescents and adults within the normative intellectual range, both self and informant versions can be used together or else the self-report version can be completed on its own.

The current study provides the first psychometric evaluation of the RBQ-3. We test its validity in one population sector, autistic adults without intellectual disability. The difficulties of this group when seeking a diagnosis have been well documented [24, 25]. The RBQ-3 could help streamline assessments for professionals and potentially guide individuals onto the appropriate assessment pathway.

### RBQ-3: background and development

The RBQ-3 questionnaire has its origins in the Diagnostic Interview for Social and Communication Disorders (DISCO) [26]. The DISCO is a semi-structured clinical interview designed for use with individuals of all ages and is widely used in the assessment of DSM-5 autism diagnosis [18, 27]. The interview includes more than 50 RRB items (see [28]) including a subset of 25 sensory items [29], which has offered significant scope for questionnaire development. Initial selection of DISCO RRB items was part of the development of a questionnaire for a large-scale general population study in the north-east of England [30, 31]. The RBQ-2 [16] was subsequently validated with a community sample of infants and children [16, 32, 33] and later with 2–17 year old autistic children [34].

Subsequently, in response to the need by autistic adults for a self-report measure, the RBQ-2A [17] was introduced and was almost identical in content to the RBQ-2. Across studies of the RBQ-2 and RBQ-2A, psychometric evaluation showed group discrimination and good internal consistency in both autistic and non-autistic samples. For the RBQ-2A, convergent validity was also found with the Autism Spectrum Quotient (AQ; [35]), [17, 36], and with the Comprehensive Autistic Trait Inventory (CATI; [37]) [38]. Additionally, the RBQ-2 is sensitive to longitudinal age changes in children (from 15 to 77 months; 33), and the RBQ-2A to cross-sectional age differences in adults (above and below age 50; 36).

Principal component and factor analysis studies of the RBQ-2 and RBQ-2A in both children and adults have demonstrated their utility to capture fine-grained subdomains of RRBs [16, 17, 33, 34, 36, 38]. A robust and stable two-factor solution has consistently emerged across studies. These include a repetitive motor and sensory behaviours factor (RSMB), including rocking, repetitive hand/finger movements, spinning, sensory reactivity, and an insistence on sameness (IS) factor, including playing the same music, game or video and insisting that daily routines or objects remain the same. This two-factor model represents the most parsimonious factor solution across RBQ-2 and RBQ-2A research [17, 32, 34, 36] and was adopted for the current study of the RBQ-3. Studies using other methods such as the Autism Diagnostic Interview (ADI-R; 39) and RBS-R [14] have also reported RSMB and IS subtypes [40–43], and a recent systematic review confirms their consistency across methods and populations [19].

### The current study

The overall aim of the research was to provide initial psychometric evaluation of the self- and informant-report versions of the RBQ-3 for use in clinical practice and research. Our approach encompassed two studies focussing on a clinical sample of adults referred for an autism diagnostic assessment (Study 1) and an online general population study that included autistic and non-autistic adults (Study 2). Across these studies, we tested the psychometric properties of the RBQ-3 using different population samples, different methods, and a range of measurement testing. In Study 1, we tested cross-informant reliability and convergent validity with a clinical interview conducted by clinicians, who were blind to the RBQ-3 content for more than half of their assessments. In Study 2, the research was online and we compared adults with and without an autism diagnosis using the self-report RBQ-3. Both studies also tested scale reliability. Study 1 additionally tested the effects of age and gender on RBQ-3 responding, while Study 2 explored the effects of age, sex and cognitive ability.

#### Study 1: cross-informant reliability and convergent validity in a clinical sample

Study 1 was conducted as part of a clinical diagnostic process, focussing on a sample of adults referred for a diagnosis of autism. The first goal of Study 1 was to test the cross-informant reliability of the self and informant versions of the RBQ-3. We compared the self-report perspective of the adults attending the clinic with the perspective of a family member or long-term friend who completed the informant-report version. In testing cross-informant reliability, we predicted that if the versions are capturing the same constructs then they should correlate. Due to limited existing evidence, no specific prediction was made about whether ratings for self- and informant-report would differ in magnitude.

The second goal of Study 1 was to test convergent validity between the RBQ-3 and the DISCO-Abbreviated interview (using its DSM-5 items; [44, 45]). To increase the rigour of our approach, for more than half of the assessments the clinician conducting the DISCO interview was blind to the RBQ-3 questionnaire ratings and did not consult the questionnaire until after the diagnostic decision was made. Previous research has found significant correlations between a clinical interview (ADI-R; [39]) and the RBS-R [14, 46]. Similarly, we predicted good convergent validity of the RBQ-3, evidenced by high correlations between RBQ-3 scores and clinician diagnostic interview ratings of RRBs using the DISCO-Abbreviated. Alongside this, we predicted good divergent validity of the RBQ-3, demonstrated by lower correlations between RBQ-3 scores and clinician rated social communication abilities using the DISCO-Abbreviated. If convergent validity is found between the RBQ-3 scores and DISCO-Abbreviated RRB scores, this would provide a strong indicator of the value of the RBQ-3 to support clinicians in a health service setting. A final goal was to explore the scaling consistency of the RBQ-3; we expected good internal consistency, in line with previous versions of the RBQ [17, 34].

## Method

### Participants

Participants, aged 18–65 years, were referrals to a national specialist adult autism diagnostic service provided by one of the seven regional health boards within Wales, UK. Referrals to this specialist service are made by a range of agencies (for example, general practitioner/physician) but most individuals self-refer. In Wales, the National Health Service (NHS) adult autism diagnostic service operates separately from specialist NHS learning disability (synonymous with intellectual disability) and mental health services. These other services provide autism diagnoses if autism presents alongside either intellectual disability or mental health difficulties. The sample from the current study included no known referrals that would have been eligible for these services.

Participants comprised 138 individuals, whose data were transferred from the service in two time phases over a three and a half year period (separated due to differences in service arrangements during the COVID-19 pandemic). Of these, 110 (80%) submitted both self- and informant-report RBQ-3 questionnaires, 12 submitted self-report questionnaires only, nine submitted informant-report questionnaires only, and seven completed no questionnaires. Only participants who submitted both RBQ-3 self-report and informant-report questionnaires were included in the study (*N* = 110).

Characteristics of these 110 participants are shown in Table 1, with demographics reported for both Phase 1 and 2 data collection phases. Information about gender but not biological sex was recorded by the service. Ethnicity data was not routinely collected by the service until the second data collection phase. This resulted in 46/50 (92%) in Phase 2 reporting their ethnic group as White British.

Participants were assessed for diagnosis by a multidisciplinary clinical team of autism specialists comprising two nurse practitioners, two occupational therapists, a clinical psychologist, and a specialist speech and language therapist. Clinicians used the DISCO-Abbreviated interview [44, 45]. Fidelity between team members in using the DISCO-Abbreviated interview was assured by double coding checks, which were integrated into training and a small percentage of cases during ongoing practice. DISCO-Abbreviated item scores were transferred to the research team. Two participants had missing DISCO-Abbreviated data and of the remaining 108, 89% met the DSM-5 criteria for both Domain A (Social communication) and Domain B (Restricted and repetitive behaviours) according to the DISCO-Abbreviated DSM-5 algorithm [44]. Criteria for both these domains (A + B) must be met to produce the DISCO-Abbreviated algorithm output of DSM-5 ASD [27, 44, 45].

DISCO scores were used to guide but not determine best estimate clinical diagnosis, with the final diagnostic decisions made by two specialist nurse practitioners. These decisions were supported by clinical assessment of need, indications of symptom severity and impact, and developmental history. Data on these final clinical diagnostic decisions were not provided in the data transfer for Phase 1 (*N* = 60), and for Phase 2 these data were provided for 33 of the 50 cases, of which only 2 (6%) had a ‘not autistic’ decision. Audit data collected by the service for all referrals across the data collection phases, including the overall number of autism diagnoses, are shown in Supplementary Table S1.

**Table 1:** Study 1: Participant characteristics of autistic adults for each data transfer phase

		Phase 1 *N* = 60-	Phase 2 *N* = 50	Total *N* = 110
Age (years)	*M* (SD)	31.40 (11.02)	35.10 (11.33)	33.08 (11.27)
	Range:	18–57	20–65	18–65
Gender	Male	36 (60%)	19 (28%)	55 (50%)
	Female	22 (37%)	31 (62%)	53 (48%)
	Trans/non-binary	2 (3%)	0	2 (2%)
Autism criteria met* using DSM-5 DISCO algorithm	Yes No	53 (89.8%) 6 (10%)	43 (87.8%) 6 (12%)	96 (89%) 12 (11%)

Note: *Two participants had missing DISCO data (one in Phase 1 and one in Phase 2)

Abbreviations: DSM-5 = Diagnostic and Statistical Manual Fifth Edition [6]. DISCO = Diagnostic Interview for Social and Communication Disorders Abbreviated interview algorithm [44]

### Materials

#### Repetitive Behaviour Questionnaire-3 (RBQ-3)

The RBQ-3 consolidated the items of the RBQ-2 [16], an informant questionnaire, and RBQ-2A [17], a self-report questionnaire, to form coordinated self-report and informant-report versions for use across the lifespan (see Table S2a and S2b for a summary of the items). The content of the RBQ-2 and RBQ-2A questionnaires, which already contained the same items, remained unchanged in the RBQ-3, with ‘items’ replacing ‘toys’ across both RBQ-3 versions (as per the RBQ-2A). The questions on the self-report version were prefaced with, “Do you:”, where as the informant-report version questions were prefaced with, “Does your child, relative, or individual you know well:.” Otherwise the self and informant-report versions were identical. Respondents were asked to reflect on the RRBS that the person had shown over the last month.

The RBQ-3 scoring procedure resolved previous inconsistencies between the scaling of the RBQ-2 and RBQ-2A. The RBQ-2 has a 4-point scale for items 1–6 and a 3-point scale for items 7–20, while the RBQ-2A has a 4-point scale for items 1–6 and 13–19, and a 3-point scale for items 7–13 and 20. To date, differences between these inconsistent scaling methods have not been examined, but instead each scale has been managed by collapsing all the scores into 1–3. This means that the full range of 1–4 scaling for the RBQ-2 and RBQ-2A has not been fully represented in previous publications. The RBQ-3 unified the scaling for items 1–19 into a 4-point scale (e.g. 1 = ‘never or rarely’, 2 = ‘mild or occasional’, 3 = ‘marked or notable’, 4 = ‘serious or severe’). The four specific response options varied depending on the question, with some items also capturing additional information about the quality of the RRB (e.g. 4 = ‘serious or severe (affects others on a regular basis)’. The use of a 4-point scale instead of a 3-point scale provided greater variability and measurement sensitivity, making the upper end of the scale available for every item instead of only some items. For item 20, the original 3-point scale, which has a slightly different response format for reporting on range of self-chosen activities, was retained.

Scoring for the RBQ-3 total score and subscales relies on the use of mean scores. Items within the total score or subscale are summed and divided by number of items completed. The use of mean scores is consistent with previous scoring for RBQ-2 and RBQ-2A and allows for missing data (up to 10% of items in total scale). The mean total score can be calculated with item 20 or without item 20, as in previous publications [16]. In the current study, total scores were calculated with and without item 20 and results were almost identical, therefore our mean total score included all 20 items. Alongside the mean total score, we also calculated two factors of RSMB and IS from items 1–19, which was based on RBQ-2A subscales previously derived from autistic adults [36]. In the current study, the response scale was presented horizontally across the page to enhance readability on the printed page.

#### Diagnostic Interview for Social and Communication disorders-Abbreviated (DISCO-Abbreviated)

The DISCO [26, 47] is a 320 item semi-structured interview schedule used by a clinician with the parent or carer of individuals at all ages, or with the individual themselves. The DISCO has good inter-rater reliability [26], criterion validity [47–49] and agreement with the ADI-R [39] and Autism Diagnostic Observation Schedule (ADOS; [50]), [48, 49].

The DISCO-Abbreviated interview is based on an abbreviated set of 54 items from the full DISCO DSM-5 algorithm, 23 of which are RRB items [44]. Full details of the DISCO-Abbreviated, including its use and scoring conventions have been described elsewhere [45]. During the interview, the clinician draws on a range of information provided by the individual and/or relative, interprets the evidence in line with knowledge from specialised DISCO training, and rates each item according to the level of impairment as ‘marked’, ‘minor’ or ‘no problem.’

Clinician scoring of the DISCO-Abbreviated algorithm items in the current study followed scoring previously reported [45], with lifespan ‘ever’ codes used. The result is DISCO-Abbreviated DSM-5 scores for the Social Communication domain (A criteria) and the RRB domain (B criteria). The Social Communication domain uses 25 items made up from three subdomains: A1. social reciprocity (9 items), A2. non-verbal communication (9 items), A3. social relationships (7 items). The RRB domain uses 23 items made up from four subdomains: B1. repetitive speech, motor movements, or use of objects (6 items); B2. routines, rituals resistance to change (6 items), B3. restricted, fixated interests (4 items); and B4. Hyper or hypo-reactivity to sensory input (7 items).

Thirteen of the DISCO-Abbreviated RRB items are in the 20-item RBQ-3. Of the remaining seven items, five are drawn from the original full DISCO interview [26] but are not included in the DSM-5-Abbreviated algorithm set, and two belong to items from an earlier pre-RBQ-2 version of the RBQ questionnaire [30, 31].

### Procedure

The RBQ-3 was developed six months before data collection started. The study was approved by both the NHS Ethics Committee and the Cardiff University School of Psychology Ethics Committee in two separate reviews due to the break in data-collection.

Data were collected by the specialised adult autism diagnostic service as part of their clinic procedure, in which questionnaires were sent from the clinic by post to individuals, together with a consent form for data access for research, and information about the assessment appointment. Each adult referred to the service was sent a self-report questionnaire, and an informant-report questionnaire for their parent, spouse, other family member or long-term friend to complete. The participant brought these into the clinic appointment regardless of whether they attended accompanied by their informant, by a different person, or attended alone. The DISCO interview was carried out at the appointment; both the person attending the clinic and their informant participated.

In Phase 1, 79% of adults were accompanied to the appointment by another person; in Phase 2 it was 57.4%. During Phase 1, a blinded procedure was used in which clinicians collected the questionnaires but did not look at them until after the diagnostic decision was made.

The datasets (RBQ-3 self- and informant-report questionnaires, DISCO-Abbreviated item scores, and a cover sheet with demographic information) were anonymised before transferring to the research team during the separate phases (September 2019-March 2020 and October 2022-March 2023).

### Data analysis plan

Data analyses were carried out using SPSS Version 27 (IBM Corp, 2021). The significance level was defined as *p* < .05 and Bonferroni correction for multiple testing was applied, where appropriate. Initial data screening first explored data distribution and missing data. Distribution for all variables failed to meet assumption of normality according to the Shapiro-Wilk statistic, and normality was not improved by data transformation. Therefore, for comparisons, non-parametric analyses were used to replace parametric analyses where the pattern of significance was different, whilst all correlations used Spearman’s rho. There were no significant differences between Phase 1 and Phase 2 in patterns of missing data, data distribution, or in scores for the RBQ-3 and DISCO-Abbreviated DSM-5 domains. Therefore, all analyses are based on the combined data except for the examination of effects of clinician blindness to RBQ-3 content.

The first step of the analysis focused on scaling. Item frequencies were used to examine endorsement of the full range of the four point scale. Internal consistency of each scale was tested using inter-item Cronbach’s alpha and model reliability using McDonald’s omega correlations. In the second step of the analysis, mean total scores and mean RSMB and IS subscale scores were calculated for the RBQ-3. This stage focused on comparisons between self- and informant-report. Cross-informant reliability was tested using Spearman’s rho correlations. Differences between self-and informant report were tested using paired t-tests and Wilcoxon signed rank tests.

Convergent and divergent validity was tested between RBQ-3 and the clinicians’ DISCO-Abbreviated DSM-5 domain scores. Spearman’s rho correlations were first used to test the associations between the mean total score of RBQ-3 and the total scores for the DISCO-Abbreviated DSM-5 RRB and Social Communication domains. We then statistically tested for a difference in the strength between these two dependent correlations [51]. Further correlational analysis enabled us to explore whether the pattern of associations between the RBQ-3 and the clinician’s domain scores was similar for Phase 1 and Phase 2. This was statistically tested using Fisher’s Z test. We then examined the RBQ-3 subscale and DISCO-Abbreviated DSM-5 subdomain level, correlating RBQ-3 RSMB and IS subscale scores with DISCO-Abbreviated DSM-5 RRB subdomain scores (i.e. scores for each of the four subdomains B1-4). Additional analyses explored effects of age and gender for the mean total and subscale RBQ-3 scores.

## Results

### Data screening

Only three participants had more than one item missing on any RBQ-3 questionnaire and all three had less than 20% of items missing for each subscale. To optimise opportunities for self-informant comparisons, all cases (*n* = 110) were retained. Two of the sample had either most or all DISCO-Abbreviated data missing; consequently, the sample reduced to 108 for DISCO-Abbreviated analyses.

Endorsements across the new RBQ-3 4-point scale by self- and informant-report are summarized in item frequency tables (Supplementary Tables S2a and S2b). The fourth point of the scale was substantially endorsed by both groups. For the self-report group, 9/19 (47%) of items were rated 4 (e.g., serious, or severe) by a quarter or more of the sample. This also applied to 7/19 (37%) of items for the informant-report group. As the RBQ-2A had collapsed the ratings scores of 3 and 4, Supplementary Table S3 shows the RBQ-3 ratings of 3 and 4 collapsed together for comparison. All three supplementary tables show a higher frequency of endorsement for items in the second half of the questionnaire, which relates to routines and change. Supplementary Table S3 also enables comparison of the percentage frequency for the 13 DISCO-Abbreviated DSM-5 RRB items that were equivalent to items in the RBQ-3 questionnaire.

### Internal consistency

For the self-report version, Cronbach’s inter-item alpha coefficients were α = 0.77 or above for all scales (RSMB = 0.77, IS = 0.88, total = 0.90). McDonald’s omega coefficients, also ω = 0.77 or above, were almost identical to the alpha coefficients (RSMB = 0.77, IS = 0.87, total = 0.90). For the informant-report version, Cronbach’s alpha coefficients were, α = 0.76 or above for all scales (RSMB = 0.76, IS = 0.89, total = 0.90) and omega coefficients were ω = 0.77 or above with almost identical coefficients to Cronbach’s alpha (RSMB = 0.77, IS = 0.88, total = 0.90).

### Cross-informant reliability

Table 2 summarises the mean total RBQ-3 self-report and informant-report scores and corresponding statistics. Spearman’s rho correlations showed high levels of cross-informant reliability between self- and informant-report. Further analysis using intra-class correlations to test for self-informant agreement for each scale produced coefficients above 0.70 for all scales. Paired *t*-tests showed that the informant-report scores were significantly lower than self-report scores at the scale level.

Supplementary Tables S2a, S2b, S2c and S3 provide information of the self- and informant endorsement at the detailed item-level. Statistical mean item comparisons (paired *t* tests) showed high effect sizes according to Cohen’s d classification (> 0.8) for 16 of the 20 mean item comparisons and medium effect sizes (> 0.5) for the remaining four. However, when Bonferroni correction (0.05/20, *p* = .0025) was applied to examine items differences, the informant ratings were significantly lower than the self-ratings for only three items, including two sensory items (items 9 [smell], 10 [feel] and 13 [home]).

**Table 2:** Study 1: Mean subscales and total score for the Repetitive Behaviours Questionnaire-3 (RBQ-3) for both self- and informant-report. (*N* = 110)

	Self version	Informant version	Spearman’s rho	Paired t-test (Cohen’s d)
**RSMB (SD)**	2.25	2.05	*r*_*s*_ =0.69	*t =* 3.87
	(0.68)	(0.71)	*p* .<001	*p* .<001
				*d =* 0.53
**IS (SD)**	2.68	2.52	*r*_*s*_ =0.72	*t* = 3.13
	(0.69)	(0.75)	*p* .<001	*p*. <002
				*d =* 0.53
**Total (SD)**	2.48	2.32	*r*_*s*_ =0.71	t = 3.60
	(0.61)	(0.64)	*p* .<001	*p* .<001
				*d =* 0.47

Note: RSMB: Repetitive Sensory Motor Behaviours, IS Insistence on Sameness

### Convergent and divergent validity with DISCO-Abbreviated DSM-5 scores

To analyse convergent and divergent validity between RBQ-3 and the DISCO-Abbreviated, we carried out a series of analyses. First, Spearman’s rho correlations were run between the RBQ-3 mean total score and the DISCO-Abbreviated DSM-5 domains. For the DISCO-Abbreviated DSM-5 RRB domain score, moderately sized correlations were found for self- (*r*_*s*_=0.54, *p* < .001) and informant-report (*r*_*s*_=0.45, *p* < .001). For the DISCO-Abbreviated DSM-5 Social Communication domain, effect sizes were smaller and the correlation was only significant for informant-report (self-report: *r*_*s*_ = 0.10, *p* > .05; informant-report: *r*_*s*_ = 0.21, *p* < .03). Additional analysis indicated that the correlations with the RBQ-3 mean total score were significantly stronger for the DISCO-Abbreviated RRB domain than the DISCO-Abbreviated Social Communication domain, for both self- (*Z* = 4.63, *p* < .001) and informant-report (*Z* = 2.46, *p* < .05).

Separate analyses were conducted for Phase 1 (*n* = 60), when the clinician was blind to the RBQ-3, and for Phase 2 (*n* = 50), when the RBQ-3 scores were available. The mean total RBQ-3 self-report score significantly correlated with DISCO-Abbreviated DSM-5 RRB domain score in both Phase 1 (*r*_*s*_ =0.43, *p* < .001) and Phase 2 (*r*_*s*_ = 0.68, *p* < .001). The correlations for informant-report scores were also significant in both Phase 1 (*r*_*s*_ =0.33, *p* < .005) and in Phase 2 (*r*_*s*_ = 0.62 *p* < .001). For the DISCO-Abbreviated DSM-5 Social Communication domain score, there was no significant correlation with the mean total RBQ-3 self-report score for either Phase 1 (*r*_*s*_ =0.05, *p* > .05) or Phase 2 (*r*_*s*_ =0.19, *p* > .05); or with the mean total RBQ-3 informant- report for either Phase 1 (*r*_*s*_ =0.18, *p* > .05) or Phase 2 (*r*_*s*_ =0.24, *p* > .05). Additional analysis indicated that the correlations between the RBQ-3 and the clinical scores were significantly stronger in Phase 2 (where RBQ-3 scores were available to clinicians) than Phase 1 for the DISCO-Abbreviated DSM-5 RRB domain (RBQ-3 self-report, Z = -1.87, *p* < .05; RBQ-3 informant-report, Z = -1.94, *p* = < 0.05), but not for the DISCO-Abbreviated DSM-5 Social Communication domain (RBQ-3 self-report, Z = − 0.72, *p* > .05; RBQ-3 informant-report, Z = − 0.32, *p* = > 0.05).

Next, we examined associations between RBQ-3 subscales and DISCO-Abbreviated DSM-5 RRB subdomain scores. First, for both the RBQ-3 self- and informant-report versions, we carried out two separate sets of analyses (RSMB and IS) with the four DISCO-Abbreviated DSM-5 RRB subdomain scores, B1-B4 (Bonferroni correction 0.05/4, *p* = .012). As shown in Table 3, only the DISCO-Abbreviated DSM-5 RRB subdomain scores for B1 (repetitive speech, motor movements, or use of objects) and B4 (Hyper or hypo-reactivity to sensory input) significantly correlated with RBQ-3 RSMB scores, with this pattern replicating for both self- and informant-report. However, all DISCO-Abbreviated DSM-5 RRB subdomains significantly correlated with RBQ-3 IS scores, for both self- and informant-report.

**Table 3:** Study 1: Spearman’s rho correlations between Repetitive Behaviours Questionnaire-3 (RBQ-3) subscales and the DISCO-Abbreviated DSM-5 RRB B1, B2, B3 and B4 subdomains for self- and informant-report (*N* = 108)

	Self-report		Informant-report	
	RSMB	IS	RSMB	IS
**B1**	0.31**	0.29*	0.36**	0.26*
**B2**	0.21	0.33**	0.15	0.26*
**B3**	0.17	0.29*	0.07	0.27*
**B4**	0.41**	0.47*	0.34**	0.37**

DISCO-Abbreviated = Diagnostic Interview for Social and Communication Disorders Abbreviated version [44]; RRB = Restricted and repetitive behaviours; B1 = repetitive speech, motor movements, or use of objects; B2.= routines, rituals resistance to change; B3 = restricted, fixated interests; B4 = Hyper or hypo-reactivity to sensory input; RSMB = repetitive motor and sensory behaviours; IS = insistence on sameness. *p < .012 (Bonferroni correction 0.05/4); ** *p* < .001

Finally, additional analyses revealed that age and gender did not significantly correlate with the RBQ-3 mean total or subscale scores in this sample for either self- or informant-report measures. For age, correlations were all *p* > .05, while for gender, point-biserial correlations for male/female were all *p* > .05.

## Discussion

The results from Study 1 help to validate the RBQ-3 for use in a clinical setting with adults who are able to self-report. First, consistent with research using the RBQ-2 and RBQ-2A, we found that the RBQ-3 total score and its RSMB and IS subscales had good internal consistency. Coefficients for Cronbach’s alpha and McDonald’s omega were almost identical to each other. Coefficients were also identical across the self- and informant-report questionnaire versions. Given that all items in the two versions are the same, this provides evidence of an identical level of internal reliability when the scale is used by different informants.

Moreover, our results show that the new 4-point scale has utility, with all points of the scale being used consistently by both types of respondents. We found convergence between RRB ratings of individuals referred for diagnosis (self-report) and the ratings of their relatives and long-term friends (informant-report), indicating measurement consistency and cross-informant validity of the RBQ-3. To our knowledge, only one study has previously compared self- and informant-report for a questionnaire measuring RRBs [43]. In a small sample (*n* = 30), autistic young people and adults showed a moderate correlation with the responses of their parents/caregivers for the RSMB subscale of the RBS-R (*r*_*s*_ = 0.39) but a weak correlation for the IS subscale (*r*_*s*_ = 0.12). The autistic people tended towards higher endorsement of RRBs than their parents/caregivers, although this was only significant for the RSMB subscale. The current study echoes the pattern of this earlier work.

Alongside the significant correlations between self- and informant-report for the RBQ-3 total score and subscales, we found significantly greater endorsement of RRBs for the adults attending the clinic compared to their informant. Looking beyond RRBs and within the wider context of autism research, very few studies have previously investigated the self-report of autistic adults compared to informants. Evidence is generally mixed, with suggestion of higher self-report of general autistic traits in autistic adults compared to the report of their informant, as well as no significant difference [52, 53]. Similarly, some prior research has found no significant associations between the responses of autistic people and their caregivers [52, 54], but this is not universal [53]. Contextual factors are likely to be relevant to these discrepancies. In the current study, a group of adults without intellectual disability who were on a waiting list for an autism diagnostic assessment were likely to have spent time reflecting on their autistic features. This is arguably a different population to those recruited through social media for a research study. The extent to which the inner world of RRBs that autistic people experience [55] are shared with those around them is an open question that has not been directly explored and deserves further investigation.

Importantly, we found convergence not only between the self- and informant-report RBQ-3 ratings, but also with RRBs assessed through clinical interview, even when the clinician was blind to RBQ-3 responses. We found moderate sized correlations between the mean total scores on the RBQ-3 questionnaire and the scores for the DISCO-Abbreviated DSM-5 RRB domain, for both self- and informant-report. In contrast, divergent validity was demonstrated through primarily non-significant correlations between the RBQ-3 and the DISCO-Abbreviated DSM-5 Social Communication domain scores. The dissociation between the DISCO-Abbreviated domains was further supported by evidence that the RRB domain score was significantly more strongly associated with RBQ-3 scores than the Social Communication domain score. This underlines the validity of the RBQ-3 as a specific measure of RRBs, rather than a more general correlate of autistic features.

Further interrogation of the convergent validity between the RBQ-3 and the DISCO-Abbreviated DSM-5 RRB algorithm established that the RSMB subscale of the RBQ-3 was selectively significantly associated with the RSMB-relevant subdomains of the DISCO-Abbreviated DSM-5 RRB algorithm, namely B1 (repetitive speech, motor movements, or use of objects) and B4 (hyper- or hypo-reactivity to sensory input). In contrast, the IS subscale of the RBQ-3 correlated with all four RRB subdomains of the DISCO-Abbreviated DSM-5 algorithm, rather than just the two subdomains (B2, B3) that best reflect IS behaviours. The lack of selectivity in association between the IS subscale of the RBQ-3 and DISCO-Abbreviated DSM-5 RRB subdomains needs further investigation. The RBQ-3 IS subscale contains more items than the RSMB subscale, which might account for this difference. However, sensory features have been associated with both the RSMB and IS subscales of the RBQ-2 in children [34]. This may partly explain the associations between the IS subscale and the DISCO-Abbreviated sensory reactivity subdomain but is less successful in accounting for the association with the motor movement subdomain. Given that the RBQ-3 RSMB subscale and the DISCO-Abbreviated motor movement subdomain both had low endorsement in the current study, it is possible that the pattern of associations might be different if these methods were used with children or with individuals with intellectual disability (see [45] for comparisons with a different dataset).

The moderately sized correlations between the RBQ-3 and the DISCO-Abbreviated DSM-5 RRB algorithm were comparable with the strength of correlations found in similar studies [30, 46]. These effect sizes also need to be considered in the context of the modes in which the information was obtained. For the DISCO-Abbreviated, the interview responses were determined by both the person attending the clinic and their informant, whereas they each had their own RBQ-3 questionnaire. Also, the RBQ-3 required consideration of RRBs over the recent past, whereas the DISCO-Abbreviated scores required reflection on behaviours over the lifespan.

Another important contextual factor is the clinician’s awareness of the RBQ-3 scores. Although convergent validity was observed between the RBQ-3 total mean score and the DISCO-Abbreviated DSM-5 RRB domain for both Phase 1 and Phase 2, there were differences in the strength of these associations. The correlations between the RBQ-3 and DISCO-Abbreviated DSM-5 RRB domain were significantly stronger in Phase 2, where clinicians had access to the RBQ-3 scores prior to the interview, than in Phase 1, where the RBQ-3 was not consulted in the diagnostic process. In contrast, the strength of correlations between the RBQ-3 and DISCO-Abbreviated DSM-5 Social Communication domain did not significantly differ between phases. Interpreting these data is limited given that we were not able to control for additional contextual factors, such as order effects, the impact of COVID-19, and changes in the clinician carrying out the clinical interview. Tentatively, these data indicate that clinicians are influenced by RBQ-3 scores when interviewing about RRBs using the DISCO-Abbreviated. This is not surprising, particularly given that the purpose of the RBQ-3 is to supplement and support the diagnostic process, and because 13 items in the DISCO-Abbreviated interview directly map onto RBQ-3 questions. Qualitative investigation of clinicians’ use of the RBQ-3 during the diagnostic process, and particularly during clinical interviews, would be illuminating. Meanwhile, the current results provide clear evidence of validity of the RBQ-3. Even when the clinician was blind to the content of the RBQ-3 in Phase 1, the correlations with the DISCO-Abbreviated DSM-5 RRB Domain were in the moderate range.

Taken together, these results suggest that when adults are referred for an autism diagnosis, their report of RRBs can be reliable across measures and across informants.

### Study 2: internal consistency in an online research sample of autistic and non-autistic adults

Study 2 provided a follow-up to Study 1, using the self-report version of the RBQ-3 with an online research sample. The main aim of Study 2 was to test the scale reliability of the RBQ-3 self-report version in diagnosed autistic and non-autistic adults. This study design complemented that of Study 1, as the current sample included autistic adults who had experienced a range of diagnostic pathways and were not all diagnosed in adulthood. In line with consistent evidence that RRBs are elevated in autistic people compared to non-autistic people [1], including previous research with autistic adults using the RBQ-2A [17] and recent research using the RBQ-3 in its Spanish translation [56], we expected to find significant differences in RBQ-3 scores between autistic and non-autistic adults. The study also enabled further analysis of the effects of sex, age and cognitive ability on RBQ-3 scores and, like Study 1, reported response patterns of endorsement and scaling distribution for each RBQ-3 item.

## Method

### Participants

One-hundred and fifty one autistic (age 18–63, *M* = 30.51, *SD* = 9.59) and 151 non-autistic (age 18–67, *M* = 31.70, *SD* = 10.62) adults living in the UK participated in an online study. All participants were recruited via Prolific (www.prolific.com), having previously participated in studies on autism. The autistic group had previously provided information about their diagnosis, including age of diagnosis, type of diagnosis (e.g. Asperger Syndrome; ASD), and the professions of the clinician(s) who made the diagnosis (e.g. clinical psychologist, psychiatrist). This information was confirmed during the current study. Table 4 shows the participant characteristics of the two groups. Within the autistic group, 46% reported having received an autism diagnosis in childhood. The non-autistic participants confirmed that they did not have a clinical autism diagnosis or suspect that they were autistic. Supplementary Table S4 shows additional diagnoses that the groups reported, together with further information on how diagnostic status was established.

To be consistent with previous RRB research, and to enable replication and groupwise-matching, participants were matched on sex rather than gender between groups. Additionally, as 15 participants in the autistic group identified their gender as ‘other’, this also prevented gender-based matching. The autistic and non-autistic groups were also matched on age and general cognitive ability. Participants completed the AQ-10 [10] to provide a general measure of autistic traits. As expected, autistic traits, as measured by the AQ-10, were higher in the autistic group (*t* (300) = 10.70, *p* < .001).

**Table 4:** Study 2: Participant characteristics of autistic (*N* = 151) and non-autistic (*N* = 151) groups

		Autistic	Non-autistic
**Age (years)**	*M* (SD)	30.51 (9.59)	31.70 (10.62)
	Range:	18–63	18–67
**Sex**	Male	73 (48.3%)	73 (48.3%)
	Female	78 (51.7%)	78 (51.7%)
**Age at diagnosis**	*M* (SD)	20.62 (11.52)	/
	Range Number with childhood diagnosis (age 3–17 years)	3–53 69 (46.0%)	/ /
**AQ-10 Score**	*M* (SD)	7.07 (2.12)	2.61 (2.02)
	Range Score of 6 or above (cut-off)	2–10 113 (75.3%)	0–10 12 (8.0%)
**Location**	England	129 (85.4%)	134 (88.7%)
	Scotland	14 (9.3%)	10 (6.6%)
	Wales	7 (4.6%)	5 (3.3%)
	N. Ireland	1 (7%)	2 (1.3%)
**ICAR score**	*M* (SD)	8.46 (3.68)	8.05 (3.26)
	Range	0–16	1–16

AQ-10 = Autism Spectrum Quotient-10; ICAR = International Cognitive Ability Resource

### Materials and procedure

#### Repetitive Behaviours Questionnaire-3 (RBQ-3)

Details of the RBQ-3 are provided in Study 1. For this study, questionnaire items and responses were presented vertically and were completed online rather than in paper format. Like Study 1, mean total scores were calculated with and without item 20 and results were almost identical; consequently, the mean total with item 20 was reported for all results.

#### International Cognitive Ability Resource (ICAR)

The International Cognitive Ability Resource (ICAR; 57) contains items testing matrix reasoning, three-dimensional rotation, verbal reasoning, and letter and number series. This cognitive measure is suited for online use and is strongly correlated with in-person tests, including the Wechsler Adult Intelligence Scale [57]. It has previously been used in research with autistic adults (e.g.); [58, 59]. We used the 16-item ICAR Sample Test [57]; scores ranged between 0 and 16 with higher scores indicating better performance.

### Procedure

The study was approved by Cardiff University’s School of Psychology Ethics Committee. Participants completed the questionnaires and assessments described above, along with two questionnaires not relevant to the current study. Each participant was paid £10 per hour for their participation.

### Data analysis plan

Data screening was conducted to explore data distribution and missing data. The RBQ-3 variables failed to meet assumptions for normality according to the Shapiro-Wilk test, and therefore the same approach was taken to parametric and non-parametric analyses as for Study 1. As in Study 1, Bonferroni correction was applied to adjust for repeat testing, where appropriate.

First, we focused on data screening and scaling, examining the endorsement of the four point scale and testing internal reliability of RBQ-3 total score and subscales with Cronbach’s alpha and McDonald’s omega correlations. In the second step, RBQ-3 total and subscale scores were compared for autistic and non-autistic participants. Subsidiary analyses were then run to examine the effect of sex, age and cognitive ability on RBQ-3 scores.

## Results

### Data screening and scaling

No data were missing for any of the variables except for the age at which diagnosis was made (4 missing). Supplementary Tables S5a-S5b show the pattern of responses for each questionnaire item for each group, with the percentage endorsement for each of the four points of the scale. Examination of scaling distribution showed that the full scale range was strongly endorsed by autistic individuals, with 10 items receiving a rating of 4 by 50% of the group. For non-autistic participants, endorsement of the highest point of the scale was much lower (0–3% of the group across items). The number of participants who scored 3 or 4 for each item was compared with a previous sample of autistic adults who completed the RBQ-2A [36] and is presented in Supplementary Table S6. Additional analysis found that using the 4-point scale led to significantly higher overall scores than the 3-point scale for all scores, except for the non-autistic group on the RSMB subscale where the effect was marginal (see Supplementary Table S7).

### Internal consistency

For the autistic group, Cronbach’s alpha correlation coefficients were: α = 0.69 for RSMB, α = 0.85 for IS and α = 0.89 for total score. McDonald’s omega coefficients were almost identical, ω = 0.69, 0.85 and 0.88 respectively. For the non-autistic group Cronbach’s coefficients were, α = 0.68 for RSMB, α = 0.76 for IS and α = 0.82 for total score, and omega coefficients were slightly higher (ω = 0.70, 0.76 and 0.81 respectively).

### Group comparison

Mean and median scores for the RBQ-3 mean total score and subscales for each group are reported in Table 5. Group differences were highly significant, with large effect sizes even when Bonferroni correction was applied to adjust for repeat testing of the total score and subscales (0.05/3, *p* = .02). Group differences were found for RBQ-3 total score (*t*(300) = 18.01, *p* = .001, *d* = 2.07), RSMB (*t*(300) = 14.72, *p* < .001, *d* = 1.69) and IS (*t*(300) = 17.12, *p* < .001, *d* = 1.97).

**Table 5:** Study 2: Repetitive Behaviours Questionnaire-3 (RBQ-3) means, SDs, medians and IQRs for total mean score and repetitive motor and sensory behaviours (RSMB) and insistence on sameness (IS) subscales

	Autistic			Non-autistic	
	Mean (SD)	Median (IQR)		Mean (SD)	Median (IQR)
**RSMB**	2.07 (0.57)	2.0 (0.84)		1.29 (0.33)	1.17 (0.33)
**IS**	2.28 (0.59)	2.36 (0.91)		1.36 (0.30)	1.27 (0.45)
**Total**	2.18 (0.51)	2.25 (0.80)		1.35 (0.25)	1.30 (0.35)

### Association between RBQ-3 scores and age, cognitive ability, and sex for each group

Table 6 summarises the associations between the RBQ-3, age and cognitive ability for both the autistic and non-autistic groups, with additional associations with age of diagnosis shown for the autistic group. For the autistic group, there were no significant correlations between the RBQ-3 total score or subscales and age, cognitive ability or age of diagnosis. For the non-autistic group, there was no association between the RBQ-3 and cognitive ability but the RBQ-3 total score and RSMB subscale were both significantly associated with age, where a lower endorsement of RRBs was associated with greater age, even accounting for Bonferroni correction (0.05/3, *p* = .02). Although the age of diagnosis did not correlate with the RBQ-3 scores for the autistic group, it was significantly associated with age and cognitive ability. Autistic adults who received a diagnosis at an older age were also older at the time of participation and had higher cognitive ability. Supplementary Table S8 shows the scores on the RBQ-3 for both groups split by sex. No sex differences were found for the RBQ-3 total score or subscales in the non-autistic group. In the autistic group, effects were not significant when Bonferroni correction (*p* < .02) was applied.

Given possible differences between those diagnosed in childhood compared to adulthood, a final analysis compared scores on the RBQ-3 for the autistic group split by those diagnosed below the age of 18 years and those diagnosed at age 18 and above. No significant differences were found in these mean comparisons, including when cognitive ability was co-varied in an ANCOVA (all *p* > .05).

**Table 6:** Study 2: Spearman’s rho correlations between the Repetitive Behaviours Questionnaire-3 (RBQ-3) and age, cognitive ability (measured by the International Cognitive Ability Resource; ICAR), and age of autism diagnosis (autistic group only)

		RBQ-3: RSMB	RBQ-3: IS	RBQ-3: Total	Age	Cognitive ability
**Autistic**	**RBQ-3: IS**	0.61**				
	**RBQ-3: Total**	0.80**	0.95**			
	**Age**	− 0.13	0.05	0.01		
	**Cognitive ability**	0.11	0.02	0.05	0.06	
	**Age at diagnosis**	− 0.02	0.13	0.10	0.68**	0.28**
**Non-Autistic**	**RBQ-3: IS**	0.46**				
	**RBQ-3: Total**	0.71**	0.93**			
	**Age**	− 0.34**	− 0.17†	− 0.21*		
	**Cognitive ability**	0.14	− 0.01	0.05	− 0.11	

RSMB = repetitive motor and sensory behaviours; IS = insistence on sameness. ** *p* < .001; * *p* < .05; †*p* = .04 (not significant at the Bonferroni corrected level of *p* < .02)

## Discussion

The results of Study 2, which included autistic and non-autistic adults recruited from the general population, support the findings of our initial study and endorse the use of the RBQ-3 self-report questionnaire as a reliable measure of RRBs for adults who are able to self-report. The lack of a significant sex difference for either group replicated previous findings using the RBQ-2A [36], although the females in the autistic group tended towards scoring more highly than the males. However, whereas previous research found a negative correlation between age and the RBQ-2A RSMB subscale in an autistic sample [36], we found a negative correlation between age and the RSMB subscale and RBQ-3 mean total score in our non-autistic group only.

This study also supported changes made to scaling in the design of the RBQ-3, indicating that the fourth point of the scale (serious/severe) is seldom endorsed by individuals who do not have a diagnosis of autism. We also found that the there was a significant difference in the mean RBQ-3 scores between the autistic and non-autistic groups. This replicates a previous finding using the Spanish translation of the RBQ-3 in a smaller sample [56], as well as research using the RBQ-3’s predecessor, the RBQ-2A [17].

## General discussion

We tested the psychometric properties of the 20-item RBQ-3, an RRB questionnaire that includes self-report and informant-report versions enabling use across the lifespan. We found that its measurement quality is good when used in an adult autism diagnostic service (Study 1), where both self- and informant-report data were collected, and within a general population sample of autistic and non-autistic adults (Study 2). Additionally, we provided the first published evidence that an RRB questionnaire is consistent with clinicians’ ratings of RRBs based on a semi-structured DSM-5 interview. We also found specific associations between subscales of the RBQ-3 and the clinically-derived DSM-5 RRB subdomains, which has not been reported in previous research.

### Implications for the use of the RBQ-3 in clinical settings and future research

RRBs exist dimensionally in the population, but their presence contributes to the categorical diagnostic decision of autism. This apparent contradiction reflects that autistic people have RRBs at the extreme end of the continuum. However, for an autism diagnosis an individual also needs to show high levels of other behaviours. In addition, clinical judgement is needed to assess severity and the need for support. Therefore, a person’s RRB profile is not sufficient for categorising them as autistic, and cut-off criteria are not appropriate for this purpose. Even self-report questionnaires that do represent the range of autism traits (e.g. AQ-10) show mixed findings in predicting diagnosis in adult services [60–63], and the consensus from these studies is that such questionnaires should not be used on their own to determine a diagnosis [62]. Furthermore, the use of a cut-off score does not encourage exploration of the specific pattern of RRBs. Therefore, we suggest the RBQ-3 be used to supplement rather than replace any part of a standard assessment. The RBQ-3 does offer ratings related to the impact on self and others (e.g. “affects others on a regular basis”, and “will not tolerate any changes”), but the clinician’s assessment will also take account of the individual’s needs, the context, developmental history, and points of evidence not accessible in a questionnaire.

Although brief questionnaires should not be used to determine diagnostic decision-making, they can support a comprehensive understanding of a person’s behaviours and characteristics. Indeed, RRBs are not well captured in diagnostic observational measures and not all diagnostic interviews provide the breath and depth of coverage found in targeted RRB questionnaires [13]. For example, questionnaire information about an individual’s RRBs could contribute to a categorical clinical decision if clinicians could consult questionnaires before the individual comes for diagnosis. Given the consistency between items in the RBQ-3 questionnaire and items in a clinical interview, the clinician could gain advanced knowledge of the RRB pattern. They could select specific areas to probe further in the interview that reflect the individual’s highly scored items (e.g. the pattern of higher ratings (3 and 4) compared to lower ratings (1 and 2)). In the case of children attending for diagnostic assessment, parents can feel uncertainty about the key characteristics of autism [24]. Therefore, completing questionnaires prior to assessment may also help those attending a clinic to think about relevant behaviours and characteristics and possibly feel more confident. Additionally, clinically or self-diagnosed autistic adults have cited anxiety about being able to adequately describe their traits and characteristics during a diagnostic interview [25], such that supplementation with questionnaires may alleviate anxiety and support them in describing their experiences.

The comparison of the self- and informant-reports may also be informative for a clinician, particularly in indicating in advance where there may be divergence and convergence between the person attending the clinic and the person that knows them well. For example, a pattern of divergence may indicate that either the individual or their informant may have poor insight into RRBs and their impact.

Another way that brief questionnaires such as the RBQ-3 may be of specific use in clinical practice is at the follow-up or post-diagnostic stage, where people receiving a diagnosis are often provided with a meeting to discuss their diagnosis. Evidence suggests that more needs to be done to make post-diagnostic support effective and meaningful for autistic people and their families [24, 64]. There may be opportunities to use a brief questionnaire, such as the RBQ-3, as an explicit framework within which to explore a person’s pattern of RRBs post-diagnosis. For example, the pattern of responses on the RBQ-3 could be used to create tailored post-diagnostic materials that provide information about RRBs that are relevant to the autistic person’s own experiences. Additionally, if the person attending the clinic and their informant have a different pattern of responses on the RBQ-3 then this could suggest an opportunity for exploring and recognising the nature of self-experienced RRBs. However, these suggestions are speculative and would require further research to establish their usefulness.

The pattern of self-reported RRBs across the two studies also indicate opportunities for further research. In Study 1, the scores on the DISCO-Abbreviated DSM-5 items (Supplementary table S3) showed that the clinicians’ DISCO-Abbreviated scores, although also generally lower than the RBQ-3 self-report scores, tended to be higher than the informant scores. It is possible that self-experience of symptoms in a diagnostic referral setting is more enhanced than it would be after diagnosis, and this enhanced experience is also communicated to the diagnosing clinician. Consistent with this argument, the self-report scores of the autistic adults in Study 2, who had an existing diagnosis, were lower than in Study 1, although the scores of the autistic participants in Study 2 showed a marked difference to the scores of the non-autistic group, who seldom used the top end of the 4-point scale. Of note, the age of autism diagnosis for the participants in Study 2 did not correlate with their RBQ-3 scores, nor was there a significant difference when a categorical distinction between those diagnosed in childhood or adulthood was used, suggesting against the interpretation that those diagnosed later in life may interpret or identify their RRBs differently. These patterns of results may be used to generate hypotheses about self-experience and self-perception of autistic traits that can be tested in future research designs.

In general, effects of current age on the subjective report of RRBs were not found in the current research, except for the non-autistic group in Study 2. However, age effects were reported in previous research using the RBQ-2 with children [33] and the RBQ-2A with adults [36]. Longitudinal research is needed to more accurately explore developmental changes in the pattern of RRBs. The number of longitudinal studies that extend into adulthood are limited, with existing data suggesting a reduction in RRBs in adulthood [65, 66]. As the first lifetime measure of RRBs, the RBQ-3 affords new opportunities for longitudinal research.

Finally, in terms of implications for clinical practice, use of the RBQ-3 questionnaire may be supplemented by further research that explores the clinical utility of different patterns of response (e.g. the pattern of higher vs. lower ratings across the 20 items). Future research on individual differences in RRB patterns should help enhance understanding of the dimensional characteristics of RRBs for clinical use. Additional research is also needed to follow up the self-experiences of RRBs in individuals who come for diagnosis alone and with no input from a family member or friend. The current findings are positive in showing the validity of self-reporting in relation to informant-reporting of RRBs. However, further research is needed to explore the convergence between the RBQ-3 and DISCO-Abbreviated DSM-5 RRB domain in adults who are unaccompanied, alongside broader investigation of their diagnostic outcomes.

### Wider use of the RBQ-3

An important facet of RRBs is that although they are characteristic of autism when experienced at a certain level of frequency, intensity and impact [6], they are by no means specific to autism. Indeed, they are frequently observed across the wider population [2, 16, 17, 67] and in other conditions [2–5, 22, 23, 68].

In isolating two specific subtypes (RSMB, IS) of RRBs, the RBQ-3 may be useful to researchers taking dimensional [69] and transdiagnostic [70] approaches to understanding difference. Research using the RBQ-3’s informant-report predecessor, the RBQ-2, has demonstrated the utility of the measure in capturing RRBs found in children without neurodevelopmental diagnoses but with a range of emotional, behavioural and/or cognitive difficulties [21]. Particularly, RBQ-2 scores were associated with parent-reported anxiety, as well as with emotion, conduct, hyperactivity, and peer-relation scores on the Strengths and Difficulties Questionnaire [71]. This reflects a myriad of studies that have shown that anxiety is associated with RRBs in autistic people [34, 72, 73], with RRBs thought to offer a soothing and self-regulatory function [74]. Similarly, higher scores on the RBQ-2 were associated with pragmatic language difficulties in the same sample, again reflecting an association that is also seen in autistic children [75]. Although further work is required to evidence the validity of the RBQ-3 in other populations, using measures such as the RBQ-3 to step outside of diagnostic classification, particularly from a longitudinal and developmental perspective, may enable broader questions to be asked about the presence and function of RRBs.

The potential for the RBQ-3 to support a dimensional approach to understanding differences in RRBs is an additional reason why an autism cut-off, which reduces a population into two categories, is not necessarily helpful. From a clinical or educational perspective, further research may support the usefulness of the RBQ-3 in helping better understand a non-autistic person’s profile of difference. For example, if a child is having difficulties in the classroom with the need to be flexible, an educational psychologist or teacher may find it useful to probe whether there is a broader pattern of RRBs.

### Limitations

There are some significant limitations to the interpretation of our results because the clinical and online samples included in our studies represented only a restricted section of the autistic population, adults who are able to self-report. Due to the structure of the health service that participated in Study 1, adults were referred to the diagnostic service only if they did not already qualify for services for people with intellectual disability or mental health difficulties. For the most part therefore, participants in Study 1 did not have an intellectual disability or mental health issues that would have necessitated referral to these other services. There may have been individuals who had coincidental intellectual disability or mental health conditions that were not routed to those services, but these details were not recorded in the research data.

There are other limitations in Study 1 that impede the generalisability of our results across clinical services. First, very few individuals attending the clinic in Study 1 received an alternative diagnosis or ‘non-autistic’ outcome, which is not necessarily representative of other adult services. Another limitation is that the DSM-5 interview criteria were collected using only one clinical interview, the DISCO-Abbreviated. Study 2 established that autistic adults endorse, on average, significantly more RRBs than their non-autistic counterparts. However, although the autistic adults had to confirm they had a clinical diagnosis and provide relevant information, diagnosis was not independently verified and therefore limits the findings. In summary, given the characteristics of the single, specialist diagnostic service included in Study 1 and the self-disclosing autistic sample in Study 2, the current findings need to be replicated and extended. Future research needs to include different autism and mental health services in different locations, and using different standardised diagnostic tools.

There are other limitations related to the sample selected for our research. First, although the RBQ-3 is designed to be suitable across age and ability, the current study has not included children or individuals with intellectual disability. We chose adults without intellectual disability as our first population to test as they would provide the most feasible and reliable cross-informant validity, with identical questionnaires being used. Further research is needed to establish the age and cognitive ability at which the self-report version of the RBQ-3 can be reliably given. While the findings of a strong correlation between informant-report and self-report provides some confirmation that RRBs can be represented by others, our current findings for adults in the normative intellectual range indicate than an informant’s ratings are generally lower than the ratings of the person presenting for diagnosis. The findings also indicated that self-reported information is more closely related to clinician ratings than the ratings of the relative or other person known to the participant. The lack of access to a self-completed version is therefore a barrier for individuals unable to complete these questionnaires. To enable recognition of the voices of people with intellectual disability, and of young people who can represent their experiences, it is essential to develop resources that increase the accessibility of the RBQ-3. This may include supporting Likert scale responses with visual representations or adapting wording [76]. This should be an important priority in the field, particularly as a recent meta-analysis has highlighted that repetitive motor behaviours are more prevalent in those with lower levels of intellectual ability [19]. Broader issues of representation that need to be addressed in future research include that sample used in Study 1 were primarily White British, and the ethnicity of the sample used in Study 2 was not collected.

Comment should also be made on the analyses included in the current research. Given the current sample size, factor analyses were not conducted. However, it is important to note that items included in the RBQ-3 remained the same as in the RBQ-2 and RBQ-2A. The RBQ-3 subscales used in this study were derived from a previous principal component analysis of the RBQ-2A in autistic adults [36]. In Study 2, the Cronbach’s alpha and McDonald’s omega for the RSMB subscale were marginal for the autistic group and non-autistic group, respectively (α = 0.69, 0.68; ω = 0.69, 0.70), given the usual level of an acceptable Cronbach’s alpha of 0.70 and above [77]. These reliability statistics were lower than we found for the RSMB subscale for autistic adults in Study 1 (α = 0.77, ω = 0.77), as well as lower than the RSMB subscale of the RBQ-2A in autistic adults [36] and non-autistic adults [17] (both studies α => 0.70), and the RSMB subscale of the RBS-R when completed by autistic adolescents and adults (α = 0.80) [43], and are likely to be specific to the sample population for Study 2. Nevertheless, further psychometric testing of larger samples is needed. To date, the two-factor solution for the RBQ-3 has been widely adopted as the most parsimonious subtyping summary in research studies using the RBA-2 and 2 A [17, 32, 34, 36]. However, a four-factor solution has also been documented [16, 38], and has also been observed in studies using other measures [78]. Further, a recent psychometric evaluation of RRB constructs across four general autism measures suggested a three-factor solution [13]. Therefore, an additional factor analysis of the RBQ-3 could be particularly informative once data are collected across a wider age and cognitive ability range, to test lifetime factors versus age-relevant factors. Additionally, although we reported patterns of item responses for both studies in supplementary tables, our analysis of response patterns was confined to the use of the rating scale. Further analyses comparing endorsement patterns across samples are needed.

Finally, the current study involved relatively limited involvement from the autistic community, which was partly because the RBQ-3 was a consolidation of two existing measures. However, two autistic adults gave feedback on the readability and acceptability of the materials included in Study 2. Currently, very few autism measures include meaningful input from the autistic community [37, 79], future research should better represent the autistic community in questionnaire development.

### Conclusion

The RBQ-3 is a questionnaire measuring repetitive behaviours that can be used across the lifespan. This research has established its psychometric properties with autistic adults without intellectual disability and confirmed its utility for this population in both clinical diagnostic and research settings in both its self- and informant-report versions. The results create opportunity for it to be further tested in other clinical settings across a range of neurodevelopmental, mental health and intellectual disability services.

Future research should be carried out with autistic children and adults, and their family members, across the spectra of age and intellectual ability, so that its utility for these groups can be ascertained. As RRBs are found in all populations, research should also target children and adults beyond diagnostic groupings. New work will help to gauge the age and intellectual level at which the self-report version can be reliably given and identify new ways for different groups to communicate their self-experienced RRBs. Whether the convergence of self- and informant-report is more reliable in certain groups or at certain times in the diagnostic process is another important area for focus. The use of self- and informant-report versions of the RBQ-3 will also be of benefit to researchers studying change in RRBs across childhood and/or adulthood.

### Electronic supplementary material

Below is the link to the electronic supplementary material.

Supplementary Material 1

## Data Availability

The datasets used and/or analysed during the current study are available from the corresponding author on reasonable request. The RBQ-3 questionnaire is freely available to download from the Cardiff University website: https://www.cardiff.ac.uk/psychology/research/impact/measuring-repetitive-behaviours-across-the-lifespan.
