# Publisher Correction: Measuring self and informant perspectives of restricted and repetitive behaviours (RRBs): psychometric evaluation of the repetitive Behaviours Questionnaire-3 (RBQ-3) in adult clinical practice and research settings

**DOI:** 10.1186/s13229-024-00609-1

**Published:** 2024-07-18

**Authors:** Catherine R.G. Jones, Lucy A. Livingston, Christine Fretwell, Mirko Uljarević, Sarah J. Carrington, Punit Shah, Susan R. Leekam

**Affiliations:** 1 Wales Autism Research Centre, School of Psychology, Cardiff University, Cardiff, UK; 2 Cardiff University Centre for Human Developmental Science, School of Psychology, Cardiff University, Cardiff, UK; 3 Department of Psychology, Institute of Psychiatry, Psychology and Neuroscience, King’s College London, London, UK; 4 Integrated Autism Service, Aneurin Bevan University Health Board, Pontypool, Wales, UK; 5 Department of Psychiatry and Behavioural Sciences, Stanford University, Stanford, CA US; 6 School of Psychology, College of Health and Life Sciences, Aston University, Birmingham, UK; 7 Department of Psychology, University of Bath, Bath, UK

**Correction**: ***Molecular Autism *(2024) 15:24**

10.1186/s13229-024-00603-7

Following publication of the article, it was noticed that partially uncorrected page proofs were mistakenly published. The original article has been corrected and the publisher apologises to the authors and readers for the inconvenience caused by this error.

Below is an overview of the corrections which have been implemented in the original article:

1. The affiliations were incorrectly assigned to the authors as: Catherine R.G. Jones^1^, Lucy A. Livingston^2^, Christine Fretwell^3^, Mirko Uljarević^4^, Sarah J. Carrington^5^, Punit Shah^6^, Susan R. Leekam^7^.
  The affiliation assignment was corrected to: Catherine R.G. Jones^1,2^*, Lucy A. Livingston^1,2,3^, Christine Fretwell^4^, Mirko Uljarević^5^, Sarah J. Carrington^6^, Punit Shah^7^, Susan R. Leekam^1,2^.
2. Throughout the article, all occurrences of “RBQ-2 A” were corrected to “RBQ-2A”.
3. In the last paragraph of the ‘Assessment of restricted and repetitive behaviours’ subsection, the position of the citation to references 24 and 25 was corrected from the end of the sentence beginning “The RBQ-3 could help (…)” to the end of the sentence beginning " We test its validity (…)”.
4. In the footnote of Table 3, a citation to reference 45 was corrected to a citation to reference 44.

