# Supplementary material for: Measuring self and informant perspectives of Restricted and Repetitive Behaviours (RRBs): psychometric evaluation of the Repetitive Behaviours Questionnaire-3 (RBQ-3) in adult clinical practice and research settings
Source: Mol Autism. 2024 Jun 6;15:24. doi: 10.1186/s13229-024-00603-7 (PMC11157832; doi:10.1186/s13229-024-00603-7)
Supplement: Supplementary file 1 — Supplementary Material 1 [file 13229_2024_603_MOESM1_ESM.docx]

**Supplementary Tables**

**Table S1.**

*Study 1:* *Number of assessments conducted by clinical team and number of cases transferred to the research team*

|  | Phase 1 | Phase 2 |
| --- | --- | --- |
|  | 01/09/2019 – 31/03/2020 | 01/10/2022 – 31/03/2023 |
| Number of assessments | 201 | 162 |
| Cases with diagnostic decision of not autistic | 9 | 6 |
| Not concluded  Consent not given  Withdrew  ADHD assessment required – withdrew | 1  1  2  0 | 7  1  1  1 |
| Number of cases transferred to the research team | 76 | 62 |
| Number of transferred cases with both RBQ-3 self- and informant-report returned | 60 | 50 |

RBQ-3 = Repetitive Behaviours Questionnaire-3

*Information on the number of cases transferred to research team in Study 1*

A sample of approximately 20 cases were transferred to the research team every two months during Phase 1 and Phase 2.

**Table S2a**.

*Study 1: Frequency and percentage response to the Repetitive Behaviours Questionnaire-3 (RBQ-3) self-report version*

|  | Never or rarely | | Mild or occasional/One or more times daily | Marked or notable/15 or more times daily | | Serious or  Severe/  30 or more times | |
| --- | --- | --- | --- | --- | --- | --- | --- |
|  |  | |  |  | |  | |
| 1. Arrange^a^ | 37 (33.9%) | | 48 (44.0%) | 16 (14.7%) | | 8 (7.3%) | |
|  |  | |  |  | |  | |
| 2. Fiddle^a^ | 10 (9.2%) | | 20 (18.3%) | 45 (41.3%) | | 34 (31.2%) | |
|  |  | |  |  | |  | |
| 3. Spin | 87 (79.1%) | | 13 (11.8%) | 6 (5.5%) | | 4 (3.6%) | |
|  |  | |  |  | |  | |
| 4. Rock^a^ | 41 (37.6%) | | 32 (29.4%) | 25 (22.9%) | | 4 (9.2%) | |
| 5. Pace^a^ | 41 (37.3%) | | 24 (21.8%) | 24 (28.8%) | | 20 (18.2%) | |
|  |  | |  |  | |  | |
| 6.Hand/finger | 28 (25.5%) | | 21 (19.1%) | 32 (29.1%) | | 29 (26.4%) | |
| 7.Fascination^a^ | 29 (26.6%) | | 21 (19.3%) | 32 (29.4%) | | 27 (24.8%) | |
| 8. Angles^c^ | 48 (44.0%) | | 28 (25.7%) | 24 (22.0%) | | 7 (6.4%) | |
| 9. Smell^a^ | 44 (40.4% | | 27 (24.8%) | 25 (22.9%) | | 13 (11.9%) | |
| 10. Feel^a^ | 30 (27.5%) | | 23 (21.1%) | 39 (35.8%) | | 17 (15.6%) | |
| 11. Carry^b^ | 44 (40.7%) | | 19 (17.6%) | 27 (25.0%) | | 18 (16.7%) | |
| 12. Collect^a^ | 18 (16.5%) | | 21 (19.3%) | 32 (29.4%) | | 38 (34.9%) | |
| 13. Home^a^ | 8 (7.3%) | | 24 (21.8%) | 36 (32.7%) | | 42 (38.2%) | |
| 14. Change^b^ | 16 (14.8%) | | 26 (24.1%) | 30 (27.8%) | | 36 (33.3%) | |
| 15. Routine^a^ | 8 (7.3%) | | 23 (20.9%) | 37 (33.6%) | | 41 (37.3%) | |
| 16. Redoing^a^ | 8 (7.3%) | | 15 (17.2%) | 21 (32.8%) | | 25 (39.1%) | |
| 17. TV/Music^c^ | 15 (13.8%) | | 14 (12.8%) | 44 (40.4%) | | 36 (33.0%) | |
| 18. Clothes | 21 (19.1%) | | 32 (29.1%) | 36 (32.7%) | | 21 (19.1%) | |
| 19. Food^a^ | 27 (24.5%) | | 28 (25.5%) | 27 (24.5%) | | 28 (25.5%) | |
| 20. Activities^a^* | 4 (3.6%) | 36 (32.7%) | | | 69 (62.7%) | |  |

^a^Missing=1; ^b^Missing=2; ^c^Missing=3. *Activities item has a 3-point scale for RBQ-3. Percentages given as valid percentages

**Table S2b**.

*Study 1: Frequency and percentage response to Repetitive Behaviours Questionnaire-3 (RBQ-3) informant-report version.*

|  | Never or rarely | | Mild or occasional/One or more times daily | Marked or notable/15 or more times daily | | Serious or  Severe/  30 or more times | |
| --- | --- | --- | --- | --- | --- | --- | --- |
|  |  | |  |  | |  | |
| 1. Arrange | 48 (43.6%) | | 43 (39.1%) | 9 (8.2%) | | 10 (9.1%) | |
|  |  | |  |  | |  | |
| 2. Fiddle | 17 (15.5.2%) | | 25 (22.7%) | 38 (34.5%) | | 30 (27.3%) | |
|  |  | |  |  | |  | |
| 3. Spin | 92 (83.6%) | | 13 (11.8%) | 1 (.9%) | | 4 (3.6%) | |
|  |  | |  |  | |  | |
| 4. Rock^a^ | 56 (51.4%) | | 26 (23.9%) | 16 (14.7%) | | 11 (10.1%) | |
| 5. Pace^c^ | 47 (43.9%) | | 25 (23.4%) | 16 (15.0%) | | 19 (17.8%) | |
| 6.Hand/finger^d^ | 41 (38.7%) | | 15 (14.2%) | 25 (23.6%) | | 25 (23.6%) | |
| 7.Fascination^a^ | 31 (28.4%) | | 18 (16.5%) | 35 (32.1%) | | 25 (22.9%) | |
| 8. Angles^b^ | 52 (48.1%) | | 27 (25.0%) | 20 (18.5%) | | 9 (8.3%) | |
| 9. Smell^a^ | 59 (53.6%) | | 23 (20.9%) | 22 (20.0%) | | 6 (5.5%) | |
| 10. Feel^b^ | 38 (35.2%) | | 31 (28.7%) | 31 (28.7%) | | 8 (7.4%) | |
| 11. Carry | 53 (48.2%) | | 16 (14.5%) | 23 (20.9%) | | 18 (16.4%) | |
| 12. Collect | 21 (19.1%) | | 28 (25.5% | 20 (18.2%) | | 41 (37.3%) | |
| 13. Home | 21 (19.1%) | | 18 (16.4) | 41 (37.3%) | | 30 (27.3%) | |
| 14. Change^a^ | 24 (22.0%) | | 22 (20.2%) | 34 (31.2%) | | 29 (26.6%) | |
| 15. Routine^a^ | 18 (16.5%) | | 17 (15.6%) | 40 (36.7%) | | 34 (31.2%) | |
| 16. Redoing^a^ | 14 (12.7%) | | 18 (16.5%) | 36 (33.0%) | | 41 (37.6%) | |
| 17. TV/Music^a^ | 19 (17.4%) | | 20 (18.3%) | 35 (32.1%) | | 35 (32.1%) | |
| 18. Clothes | 36 (32.7%) | | 29 (26.4%) | 23 (20.9%) | | 22 (20.0%) | |
| 19. Food | 35 (31.8%) | | 30 (22.7%) | 21 (19.1) | | 29 (26.4) | |
| 20. Activities^a^* | 5 (4.6%) | 30 (27.5%) | | | 74 (67.9%) | |  |

^a^Missing=1; ^b^Missing=2; ^c^Missing=3, ^d^Missing=4. *Activities item has a 3-point scale for RBQ-3. Percentages given as valid percentages.

**Table S2c.**

Study 1: Comparison of mean item endorsement on the RBQ-3 for self- and informant-report

|  | Self-report  *M (SD)* | Informant-report  *M (SD)* | Effect size  *(Cohen’s d)* |
| --- | --- | --- | --- |
| 1. Arrange^a^ | 1.95 (.89) | 1.83 (.93) | .78 |
| 2. Fiddle^a^ | 2.94 (.93) | 2.75 (1.02) | .88 |
| 3. Spin | 1.34 (.75) | 1.25 (.65) | .63 |
| 4. Rock^c^ | 2.03 (1.00) | 1.83 (1.03) | .91 |
| 5. Pace^c^ | 2.20 (1.15) | 2.07 (1.14) | .94 |
| 6. Hand/Finger^d^ | 2.56 (1.16) | 2.32 (1.22) | 1.07 |
| 7. Fascination^b^ | 2.53 (1.14) | 2.48 (1.13) | .86 |
| 8. Angles^d^ | 1.90 (.97) | 1.88 (1.00) | .93 |
| 9. Smell^a^ | 2.06 (1.06) | 1.76 (.95) | .94 |
| 10. Feel^c^ | 2.39 (1.04) | 2.07 (.97) | .84 |
| 11. Carry^b^ | 2.18 (1.14) | 2.06 (1.17) | .93 |
| 12. Collect^a^ | 2.83 (1.09) | 2.74 (1.16) | .86 |
| 13. Home | 3.02 (.95) | 2.73 (1.07) | .76 |
| 14. Change^c^ | 2.81 (1.06) | 2.63 (1.11) | 1.00 |
| 15. Routine^b^ | 3.02 (.95) | 2.84 (1.04) | .94 |
| 16. Redoing^a^ | 3.15 (.92) | 2.95 (1.03) | .87 |
| 17. TV/Music^a^ | 2.93 (1.00) | 2.79 (1.08) | .90 |
| 18. Clothes | 2.52 (1.01) | 2.28 (1.13) | 1.03 |
| 19. Food | 2.51 (1.12) | 2.40 (1.19) | .83 |
| 20. Activities^b^ | 2.59 (.56) | 2.63 (.57) | .56 |

^a^Missing=1; ^b^Missing=2; ^c^Missing=3; ^d^Missing=4

**Table S3**.

*Study 1: Percentage of individuals with marked or serious ratings (i.e. scoring 3 or 4) for* *Repetitive Behaviours Questionnaire-3 (RBQ-3) self- and informant-report items, compared with equivalent ratings for DISCO-Abbreviated interview items (clinician).*

|  | RBQ-3 Self (N=110) | RBQ-3  Informant  (N=110) | DISCO  Clinician  (N=108) |
| --- | --- | --- | --- |
| 1. Arrange | 22.0 | 17.3 | 69.4 |
| 2. Fiddle | 72.5 | 61.8 | 75.0 |
| 3. Spin | 9.1 | 4.5 | / |
| 4. Rock | 32.1 | 24.8 | / |
| 5. Pace | 40.0 | 32.4 | / |
| 6. Hand/Finger | 55.5 | 46.8 | 28.7 |
| 7. Fascination | 54.2 | 54.5 | 56..5 |
| 8. Angles | 28.4 | 26.6 | 35.2 |
| 9. Smell | 34.8 | 25.5 | 36.1 |
| 10. Feel | 51.4 | 35.7 | 54.6 |
| 11. Carry | 41.7 | 37.3 | / |
| 12. Collect | 64.3 | 55.5 | 67.6 |
| 13. Home | 70.9 | 64.6 | 68.5 |
| 14. Change | 61.1 | 57.8 | / |
| 15. Routine | 70.9 | 67.3 | 69,4 |
| 16. Redoing | 79.1 | 70.0 | / |
| 17. TV/Music | 73.4 | 64.2 | 65.7 |
| 18. Clothes | 51.8 | 40.9 | / |
| 19. Food | 50.0 | 45.5 | 45.4 |
| 20. Activities* | 62.7 | 67.9 | 80.6 |

DISCO-Abbreviated = Diagnostic Interview for Social and Communication Disorders Abbreviated version (Carrington et al., 2019). *Activities item has a 3-point scale for RBQ-3.

**Table S4.**

*Study 2: Additional self-reported diagnoses of the autistic (N=151) and non-autistic (N=151) groups*

|  | Autistic  *n*(%) | Non-autistic  *n*(%) |
| --- | --- | --- |
| ADHD | 26(17) | 1(1) |
| Dyslexia | 16(11) | 4(3) |
| Dyspraxia | 20(13) | 1(1) |
| Dyscalculia | 2(1) | 0(0) |
| Intellectual Disability | 7(5) | 0(0) |
| Speech/Language Impairment | 4(3) | 0(0) |
| Depression | 70(46) | 23(15) |
| Anxiety | 89(59) | 31(21) |
| Social Anxiety | 49(32) | 9(6) |
| Eating Disorders | 20(13) | 3(2) |
| Schizophrenia | 2(1) | 0(0) |
| Bipolar Disorder | 4(3) | 1(1) |
| Personality Disorders | 10(7) | 1(1) |
| Obsessive Compulsive Disorder | 18(12) | 1(1) |
| Post-Traumatic Stress Disorder | 7(5) | 0(0) |
| Other | 2(1) | 0(0) |

*Information on sample recruitment for Study 2:*

All autistic participants had a clinical diagnosis of autism spectrum disorder (ASD) based on the ICD or DSM (American Psychiatric Association, 2013; World Health Organization, 2019). Participants were diagnosed by UK based healthcare professional/s in a formal clinical setting. As part of the online research, diagnoses were confirmed at multiple time points during an initial screening process, in previous studies and at the start of the current study. Following recent studies on recruiting large samples of clinically diagnosed autistic people (e.g. 1, 2, 3), participants provided detailed information about their diagnosis (e.g., Asperger Syndrome, ASD), including specific details about the age at diagnosis and diagnosing clinician(s) (e.g., Psychiatrist). Individuals who self-identified as autistic, suspected they were autistic, or those seeking a diagnosis were not eligible to participate. Autistic participants were matched to a group of people who identified as non-autistic and who were matched on age, sex and general cognitive ability using the International Cognitive Ability Resource.

**Table S5a**.

*Study 2: Frequency and percentage response to Repetitive Behaviours Questionnaire-3 (RBQ-3) self-report version by the autistic group*

|  | Never or rarely | | Mild or occasional/One or more times daily | Marked or notable/15 or more times daily | | Serious or  Severe/  30 or more times | |
| --- | --- | --- | --- | --- | --- | --- | --- |
|  |  | |  |  | |  | |
| 1. Arrange | 56 (37.1%) | | 77 (51.0%) | 16 (10.6%) | | 2 (1.3%) | |
|  |  | |  |  | |  | |
| 2. Fiddle | 16 (10.6%) | | 44 (29.1%) | 45 (29.8%) | | 46 (30.5%) | |
|  |  | |  |  | |  | |
| 3. Spin | 111 (73.5%) | | 34 (22.5%) | 5 (3.3%) | | 1 (.7%) | |
|  |  | |  |  | |  | |
| 4. Rock | 57 (37.7%) | | 53 (35.1%) | 29 (19.2%) | | 12 (7.9%) | |
| 5. Pace | 46 (30.5%) | | 61 (40.4%) | 30 (19.9%) | | 14 (9.3%) | |
| 6.Hand/finger | 37 (24.5%) | | 56 (37.1%) | 37 (24.5%) | | 21 (13.9%) | |
| 7.Fascination | 39 (25.8%) | | 48 (31.8%) | 53 (35.1%) | | 11 (7.3%) | |
| 8. Angles | 48 (31.8%) | | 71 (47.0%) | 28 (18.5%) | | 4 (2.6%) | |
| 9. Smell | 85 (56.3%) | | 34 (22.5%) | 19 (12.6%) | | 13 (8.6%) | |
| 10. Feel | 57 (37.7%) | | 45 (29.8%) | 42 (27.8%) | | 7 (4.6%) | |
| 11. Carry | 64 (42.4%) | | 45 (29.8) | 26 (17.2%) | | 16 (10.6%) | |
| 12. Collect | 37 (24.5%) | | 49 (32.5%) | 43 (28.5%) | | 22 (14.6%) | |
| 13. Home | 18 (11.9%) | | 49 (32.5%) | 63 (41.7%) | | 21 (13.9%) | |
| 14. Change | 32 (21.2%) | | 57 (37.7%) | 39 (25.8%) | | 23 (15.2%) | |
| 15. Routine | 27 (17.9%) | | 46 (30.5%) | 53 (35.1%) | | 25 (16.6%) | |
| 16. Redoing | 17 (11.3%) | | 55 (36.4%) | 54 (35.8%) | | 25 (16.6%) | |
| 17. TV/Music | 19 (12.6%) | | 56 (37.1%) | 57 (37.7%) | | 19 (12.6%) | |
| 18. Clothes | 47 (31.1%) | | 47 (31.1%) | 45 (29.8%) | | 12 (7.9%) | |
| 19. Food | 45 (29.8%) | | 50 (33.1%) | 39 (25.8%) | | 17 (11.3%) | |
| 20. Activities* | 10 (6.6%) | 83 (55.0%) | | | 58 (38.4%) | |  |

N= 151 for all items. No missing data. *Activities item has a 3-point scale for RBQ-3.

**Table S5b**.

*Study 2: Frequency and percentage response to Repetitive Behaviours Questionnaire-3 (RBQ-3) self-report version by the non-autistic group*

|  | Never or rarely | | Mild or occasional/One or more times daily | Marked or notable/15 or more times daily | | Serious or  Severe/  30 or more times | |
| --- | --- | --- | --- | --- | --- | --- | --- |
|  |  | |  |  | |  | |
| 1. Arrange | 118 (78.1%) | | 33 (21.9%) | 0 | | 0 | |
|  |  | |  |  | |  | |
| 2. Fiddle | 74 (49.0%) | | 54 (35.8%) | 18 (11.9%) | | 5 (3.3%) | |
|  |  | |  |  | |  | |
| 3. Spin | 143 (94.7%) | | 8 (5.3%) | 0 | | 0 | |
|  |  | |  |  | |  | |
| 4. Rock | 125 (82.8%) | | 24 (15.9%) | 2 (1.3%) | | 0 | |
| 5. Pace | 117 (77.5%) | | 30 (19.9%) | 3 (2.0%) | | 1 (0.7%) | |
| 6.Hand/finger | 121 (80.1%) | | 21 (13.9%) | 9 (6.0%) | | 0 | |
| 7.Fascination | 127 (84.1%) | | 22 (14.6%) | 2 (1.3%) | | 0 | |
| 8. Angles | 120 (79.5%) | | 30 (19.9%) | 1 (0.7%) | | 0 | |
| 9. Smell | 127 (84.1%) | | 21 (13.9%) | 3 (2.0%) | | 0 | |
| 10. Feel | 114 (75.5%) | | 34 (22.5) | 3 (2.0%) | | 0 | |
| 11. Carry | 129 (85.4%) | | 19 (12.6%) | 3 (2.0%) | | 0 | |
| 12. Collect | 93 (61.6%) | | 53 (35.1) | 4 (2.6%) | | 1 (0.7%) | |
| 13. Home | 85 (56.3%) | | 53 (35.1%) | 12 (7.9%) | | 1 (0.7%) | |
| 14. Change | 101 (66.9%) | | 42 (27.8%) | 8 (5.3%) | | 0 | |
| 15. Routine | 87 (57.6%) | | 57 (37.7%) | 6 (4.0%) | | 1 (0.7%) | |
| 16. Redoing | 81 (53.6) | | 56 (37.1%) | 14 (9.3%) | | 0 | |
| 17. TV/Music | 78 (51.7%) | | 62 (41.1%) | 10 (6.6%) | | 1 (0.7%) | |
| 18. Clothes | 122 (80.8%) | | 26 (17.2%) | 1 (0.7%) | | 2 (1.3%) | |
| 19. Food | 11 (80.1%) | | 25 (16.6%) | 5 (3.3%) | | 0 | |
| 20. Activities* | 36 (23.8%) | 100 (66.2%) | | | 15 (9.9%) | |  |

N= 151 for all items. No missing data. *Activities item has a 3-point scale for RBQ-3.

**Table S6.**

*Study 2: Percentage of individuals in autistic (n=151) and non-autistic (N=151) groups scoring 3 or 4 for each item on the Repetitive Behaviours Questionnaire-3 (RBQ-3), compared with equivalent percentages for an autistic group reported by Barrett et al. (2018) for the RBQ-2A.*

|  | Study 2 | | Barrett et al. (2018) |
| --- | --- | --- | --- |
|  | Autistic | Non-Autistic | Autistic |
| 1. Arrange | 11.9 | 0 | 18.8 |
| 2. Fiddle | 60.3 | 15.2 | 63.8 |
| 3. Spin | 4.0 | 0 | 5.2 |
| 4. Rock | 27.1 | 1.3 | 27.6 |
| 5. Pace | 29.2 | 2.7 | 29.5 |
| 6. Hand/Finger | 38.4 | 6.0 | 42.0 |
| 7. Fascination | 42.4 | 1.3 | 47.2 |
| 8. Angles | 21.1 | 0.7 | 28.2 |
| 9. Smell | 21.2 | 2.0 | 22.2 |
| 10. Feel | 32.4 | 2.0 | 34.8 |
| 11. Carry | 27.8 | 2.0 | 27.3 |
| 12. Collect | 43.1 | 3.3 | 53.1 |
| 13. Home | 55.6 | 8.6 | 73.0 |
| 14. Change | 41.0 | 5.3 | 60.1 |
| 15. Routine | 51.7 | 4.7 | 63.5 |
| 16. Redoing | 52.4 | 9.3 | 73.4 |
| 17. TV/Music | 50.3 | 7.3 | 63.2 |
| 18. Clothes | 37.7 | 2.0 | 41.0 |
| 19. Food | 37.1 | 3.3 | 49.2 |
| 20. Activities* | 38.4 | 9.9 | 58.6 |

*Activities item has a 3-point scale for RBQ-3.

**Table S7**.

*Study 2: Mean Repetitive Behaviours Questionnaire-3 (RBQ-3) scores using the 3-point scale to compare to Barrett et al. (2018), which used the 3-point scale.*

|  | Study 2 | | | | Barrett et al. (2018) |
| --- | --- | --- | --- | --- | --- |
|  | Autistic | | Non-autistic | | Autistic |
|  | Mean  (SD) | Median  (IQR) | Mean  (SD) | Median (IQR) | Mean  (SD) |
| RSMB | 1.96 (.47) | 2.00 (.67) | 1.28 (.31) | 1.30 (.33) | 1.97 (.48) |
| IS | 2.17 (.48) | 2.27 (.73) | 1.36 (.29) | 1.27 (.45) | 2.36 (.41) |
| Total (incl. item 20) | 2.07 (.42) | 2.15 (.70) | 1.34 (.24) | 1.30 (.35) | - |

RSMB = repetitive motor and sensory behaviours; IS = insistence on sameness.

For the data in Study 2, the mean and median score on the 4-point scale (1-4; see Table 5 in the main manuscript) was consistently higher than on the 3-point scale (1-3; see above) for both groups Using the non-parametric Wilcoxon signed rank test, all comparisons for autistic group were significant (RSMB: *Z*= -7.67, *p*=.001, *r*=.44; IS: *Z*= -8.41, *p*=.000, *r*=.48; Total: *Z*=-8.55, *p*=.000, *r*=.49). For non-autistic group, the total mean score difference was significant (*Z*=-2.59, *p*=.01, *r*=.15), as was the IS (IS, *Z*=-3.21, *p*=001, *r* =.18). However, the difference for the RSMB subscale was not significant (*Z*=-1.90, *p*=.06, *r*=.12).

**Table S8:**

Study 2: *Means and standard deviations for Repetitive Behaviours Questionnaire-3 (RBQ-3) subscales and total score, presented for each group separately by sex.*

|  |  |  | Autistic |  |  | Non-Autistic | | |
| --- | --- | --- | --- | --- | --- | --- | --- | --- |
|  |  | Male | Female | All |  | Male | Female | All |
| RSMB | *M* | 2.03 | 2.11 | 2.07 |  | 1.30 | 1.27 | 1.29 |
|  | *SD* | .60 | .54 | .57 |  | .35 | .313 | .33 |
| IS | *M* | 2.18 | 2.38 | 2.28 |  | 1.37 | 1.35 | 1.36 |
|  | *SD* | .52 | .64 | .59 |  | .28 | .314 | .30 |
| Total Score | *M* | 2.09 | 2.26 | 2.18 |  | 1.36 | 1.33 | 1.35 |
|  | *SD* | .47 | .53 | .51 |  | .26 | .251 | .25 |

RSMB = repetitive motor and sensory behaviours; IS = insistence on sameness.

**References**

1. Taylor EC, Livingston LA, Clutterbuck RA, Callan MJ, Shah P. Psychological strengths and well-being: Strengths use predicts quality of life, well-being and mental health in autism. Autism. 2023;27(6):1826-39.

2. Taylor EC, Farmer GD, Livingston LA, Callan MJ, Shah P. Rethinking fast and slow processing in autism. Journal of Psychopathology and Clinical Science. 2022;131(4):392.

3. Farmer GD, Baron-Cohen S, Skylark WJ. People with autism spectrum conditions make more consistent decisions. Psychological Science. 2017;28(8):1067-76.
